# Minimally invasive percutaneous cystostomy with ureteroscopic pneumatic lithotripsy for calculus in bladder diverticula

**DOI:** 10.3892/etm.2013.1047

**Published:** 2013-04-03

**Authors:** SI-PING GU, ZHI-YUAN YOU, YUNTENG HUANG, YI-JIN LU, CAOHUI HE, XIAO-DONG CAI, XIAO-MING ZHOU

**Affiliations:** 1 Department of Minimally Invasive Surgery, Shishi City Hospital, Shishi, Fujian 362700;; 2 Department of Urology, Xinhua Hospital, Shanghai Jiaotong University School of Medicine, Shanghai 200092;; 3 Department of Minimally Invasive Surgery, Wanxiang Minimally Invasive Hospital, Quanzhou, Fujian 362000;; 4 Micro-Invasive Surgery Center, First Hospital Affiliated to Guangzhou Medical University, Guangzhou, Guangdong 510120, P.R. China

**Keywords:** minimally invasive, percutaneous cystostomy, calculus, bladder diverticulum, pneumatic lithotripsy

## Abstract

The aim of this study was to investigate the effectiveness of minimally invasive percutaneous cystostomy with ureteroscopic pneumatic lithotripsy for treating calculus in bladder diverticula. Percutaneous cystostomy with ureteroscopic pneumatic lithotripsy was performed on six elderly male patients with calculi in bladder diverticula, who could not be treated with transurethral ureteroscopic lithotripsy. The stones were successfully removed from all patients, with no complications such as bladder perforation, rupture, urethritis or cystitis. The surgery time was 15–60 min, with an average time of 32 min. Postoperative ultrasound or X-ray examination showed no stone residues and the bladder stoma healed well. No recurrent stones were detected in the follow-up of 3–24 months (average, 16 months). Minimally invasive percutaneous cystostomy with ureteroscopic pneumatic lithotripsy is a safe, efficient and easy treatment for calculus in bladder diverticula. This method provides a new clinical approach for lithotripsy and we suggest that it is worthy of wider use.

## Introduction

Calculus in diverticulum of the bladder is a rare clinical disease. In elderly males, secondary bladder diverticula often occur in the bottom of the bladder or the upper back of the bilateral trigone (1–3). As there is an angle between the body of a cystoscope or ureteroscope and the diverticulum, the stones are difficult to remove by transurethral endoscopic lithotripsy. Consequently, open surgery is often performed to remove the stones, leading to greater surgical trauma (4). In the present study, percutaneous cystostomy with ureteroscopic pneumatic lithotripsy was performed on six elderly male patients with calculi in bladder diverticula between 2005 and 2011 and the effectiveness of the treatment was investigated.

## Materials and methods

### General data

Six elderly male patients with calculi in bladder diverticula were enrolled in the present study. The patients were aged 56–80 years, with an average age of 71 years. There were 1–5 stones in each patient (average, 2.3 stones). The average diameter of the stones was 1.63±0.72 cm. Three patients were preoperatively diagnosed with bladder diverticula combined with diverticular stones. One patient was preoperatively diagnosed, using kidney, ureter and bladder X-ray (KUB), with bladder stones, which are not detected by transurethral lithotripsy. The diverticular stones were confirmed by ultrasound or X-ray examination. Two patients were diagnosed with a combination of prostatic hyperplasia, bladder stones and bladder diverticula. During transurethral lithotripsy, the stones moved to the diverticulum and could not be removed. Consequently, percutaneous cystostomy was performed. Transurethral resection of the prostate (TURP) was simultaneously performed on four patients. The patients were treated in the Department of Minimally Invasive Surgery, Shishi City Hospital, Shishi, China. The study was approved by the ethics committee of Shishi City Hospital. Written informed patient consent was obtained from the patient.

### Apparatus and materials

The apparatus and materials used were as follows: APL-II intracavity perfusion pump, APL pneumatic lithotripsy machine (EMS Electro Medical Systems SA, Nyon, Switzerland), Wolf 8/9.8 F ureteroscope (Richard Wolf GmbH, Knittlingen, Germany), Cook fascial dilator (F8–F22) (Cook Medical, Bloomington, IN, USA), 18G renal puncture needle, Urovision 0.035-inch zebra guidewire and Peel-Away sheath (F16–F22) (Urovision GmbH, Bad Aibling, Germany).

### Surgical methods

Under epidural anesthesia, the modified lithotomy position (head elevation, 15°) was used for surgery. The nephrostomy bag was fixed at the pubis position. The stones were not successfully removed by transurethral ureteroscopic lithotripsy, so percutaneous cystostomy was performed. After filling the bladder with saline, an 18G renal puncture needle was used to puncture the bladder 1–2 cm above the pubic symphysis. The puncture needle core then was drawn, leading to the appearance of a flow of urine, and a zebra guidewire was then inserted. A skin incision of 0.5–0.6 cm was made along the puncture needle and the fascial dilator was then used to gradually expand the incision to F18 (F22 for stones too large for F18) along the guidewire. The Peel-Away sheath was positioned to construct a stone removal passage. The Wolf 8/9.8 F ureteral endoscope was placed in the diverticulum and pneumatic lithotripsy was performed. The small stones were flushed out through the Peel-away sheath using saline perfusion and the larger stones were removed with lithotomy forceps. After all the stones had been removed, the F14 balloon urinary catheter was retained as a cystostomy tube. For patients also suffering from prostate hyperplasia, TURP was performed simultaneously. Postoperative ultrasound or X-ray examination was performed. Finally, the cystostomy tube and urinary catheter were removed simultaneously.

## Results

The stones were successfully removed from all patients, with no complications such as bladder perforation, rupture, urethritis or cystitis. The surgery time was 15–60 min, with an average time of 32 min. Postoperative ultrasound or X-ray examination showed no stone residues. During the 3–24-month follow-up (average, 16 months), all patients had unobstructed urination, with no urethral stricture and no recurrent stones were detected.

### Typical case 1

The patient, a 70-year-old male, was treated for gross hematuria accompanied by voiding difficulty for two months and was examined with ultrasound and KUB, which revealed a bladder diverticulum with bladder calculus (three calculi). The patient was treated with minimally invasive percutaneous cystostomy with ureteroscopic pneumatic lithotripsy. After surgery, the urination of the patient was smooth and no gross hematuria was observed.

**Figure 1:**
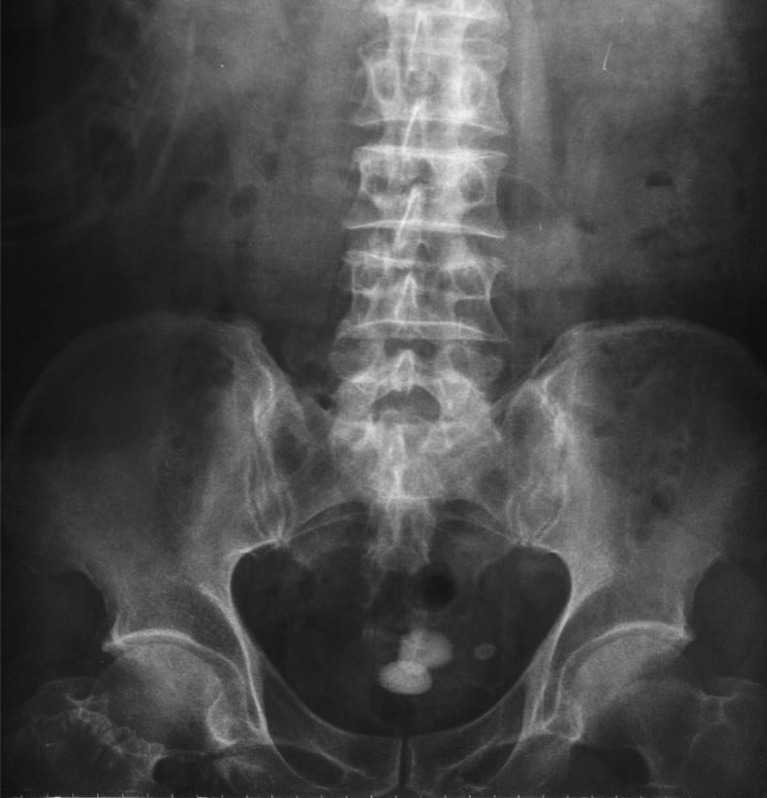
Preoperative KUB of a patient (case 1) with bladder diverticulum accompanied by bladder calculus. KUB, kidney, ureter and bladder X-ray.

**Figure 2:**
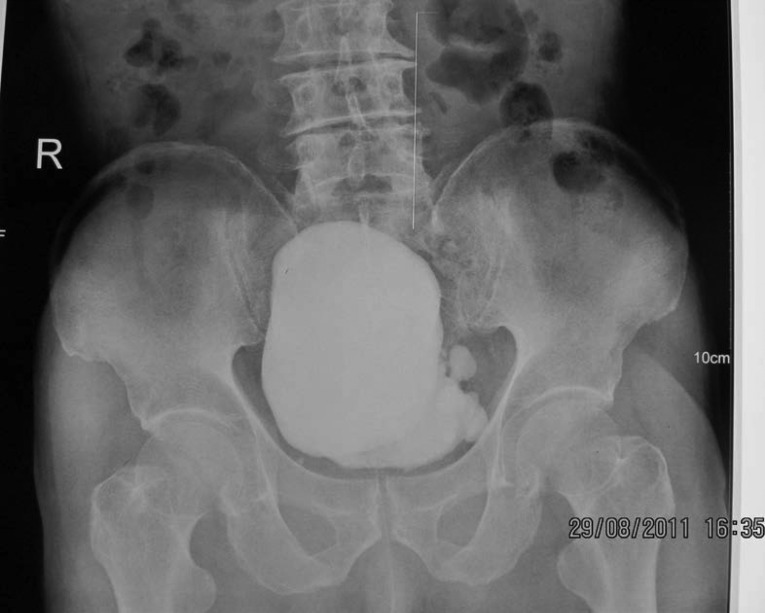
Preoperative radiography results of a patient (case 1) with bladder diverticulum accompanied by bladder calculus.

**Figure 3:**
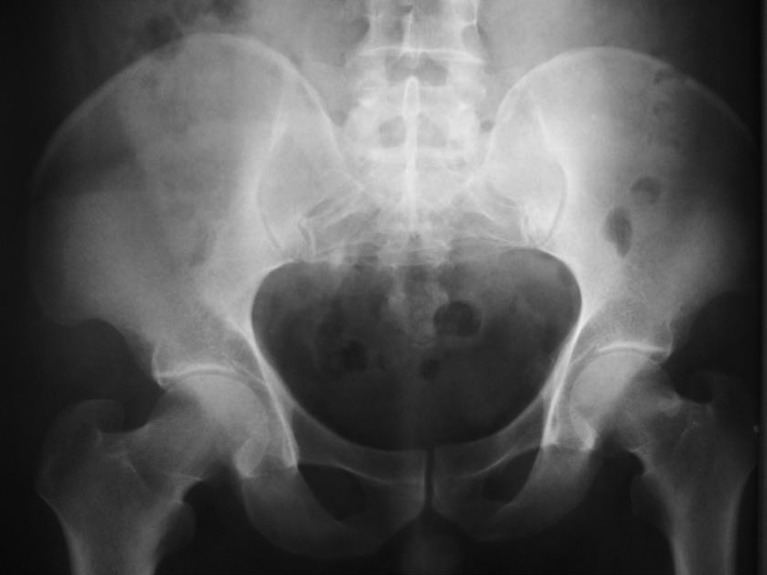
Postoperative pelvic X-ray results of a patient (case 1) with bladder diverticulum accompanied by bladder calculus.

Fig. 1 shows the preoperative KUB of the patient with diverticular bladder calculus which indicated that multiple calculi existed in the bladder. In addition, the preoperative radiography results of the patient indicated that the diverticulum existed at the top on the left lateral wall of the bladder (Fig. 2). The postoperative pelvic X-ray results of the patient indicated that the calculi in the bladder diverticulum and bladder had been completely removed (Fig. 3).

## Discussion

A bladder diverticulum is a hernia formed by urothelium penetration into the muscularis propria of the bladder wall. It is caused by the protrusion of the bladder wall from the bundle of detrusor muscle in the bladder due to congenital bladder wall weakness, lower urinary tract obstructions and increased intravesical pressure. Bladder diverticula are divided into congenital and secondary types. Congenital bladder diverticula often occur in male children, with an incidence of ∼1.7% (1–3) and are usually beside or behind the ureteral orifice. These diverticula are mostly large and solitary. As observed by an endoscope, they are located at the smooth bladder wall, with no evident trabeculation. Secondary bladder diverticula mostly occur in elderly males (>60 years), with an incidence of 1–6% in patients with a history of prostatic hyperplasia (1). Bladder outlet obstruction and urethral stricture induced by prostatic diseases are the main causes of these diverticula. There are often multiple diverticula, with evident trabeculation under a cystoscope. Iatrogenic factors may also cause a bladder diverticulum to form. The insufficient closure of a muscle layer after bladder incision may lead to weakening of the suture position, resulting in bladder diverticulum formation. In addition, lower extremity venous congestion may also result in bladder diverticulum formation (4,5).

Calculus in diverticulum of the bladder is reported less often in the clinic, particularly for treatment using minimally invasive endoscopic lithotripsy. There is no significant clinical manifestation of this disease, although urinary infection, hematuria and prostatic hyperplasia are clinical symptoms. This disease may be incidentally detected in B-scan ultra-sonography, KUB and cystoscopic examination. Calculus in diverticulum of the bladder may be diagnosed due to inguinal hernia caused by the bladder diverticulum and spontaneous bladder diverticulum rupture (6–9). In the present study, three cases were treated due to hematuria and the bladder diverticulum stones were confirmed by preoperative KUB, ultrasound examination and cystoscopy. One case was preoperatively diagnosed with bladder stones, which are not detected by transurethral lithotripsy and the diverticular stones were confirmed by ultrasound or X-ray examination. The remaining two cases had a combination of prostatic hyperplasia, bladder stones and secondary bladder diverticula. During transurethral lithotripsy, the stones moved to the diverticulum and could not be removed.

Congenital and secondary bladder diverticula do not require treatment if there are no clinical symptoms. Otherwise, diverticulum resection should be performed. For certain patients with a bladder diverticulum, intradiverticular urine retention, urinary salt deposition, infection and chronic inflammatory stimuli lead to the formation of diverticular stones, resulting in symptoms such as repeated stimulation, obstruction and even carcinogenesis (10–12). Consequently, bladder diverticulum stones should be removed as quickly as possible. For patients in whom prostatic hyperplasia is the cause of the diverticulum and stones, the prostatic obstruction should be treated simultaneously. This contributes to the treatment of secondary bladder diverticula and prevents stone occurrence (13,14).

Bladder diverticulum stones are usually removed by open surgery, with the occasional use of laparoscopic diverticulum excision (5,15,16). Open surgery not only causes larger surgical trauma, but also results in complications such as ureteral injury and stricture. In addition, other complications, such as urinary tract infection, bleeding, postoperative urinary extravasation, urinary fistula and intestinal injury, may not be completely avoided. Among elderly male patients, the bladder diverticulum often occurs in the bottom of the bladder or the upper back of the bilateral trigone. As there is an angle between the body of a cystoscope or ureteroscope and the diverticulum, the stones are difficult to remove by transurethral ureteroscopic lithotripsy. Consequently, open surgery is often performed.

In the present study, the principle of minimally invasive percutaneous nephrostomy technology was investigated. Minimally invasive percutaneous cystostomy was used to treat bladder diverticulum stones and a satisfactory result was obtained. According to the present results and associated literature (6,14,15), the reported experiences of percutaneous cystostomy with ureteroscopic pneumatic lithotripsy are as follows (14,15,17,18): i) The distance between the bladder stoma and bladder cavity is short and straight and a ureteroscope may easily pass in and out the bladder, with no angle between the ureteroscope body and diverticulum. Consequently, the stones are easily identified and removed; ii) the placement of a Peel-Away sheath maintains the low intravesical pressure, avoiding urinary extravasation and bladder rupture. Simultaneous TURP reduces the associated syndromes; iii) the stone removal and crushing are performed simultaneously under saline perfusion, which shortens the surgery time; iv) larger bladder stones may be simultaneously treated; v) this procedure does not have high equipment requirements and may be performed in hospitals with pneumatic lithotripsy, ultrasonic lithotripsy or holmium laser lithotripsy machines. vi) for certain high-risk patients without anesthetic tolerance, this surgery may be performed under conditions of basal anesthesia combined with local infiltration anesthesia; vii) this procedure has advantages of simple surgery, a short learning curve and a wide application range.

Minimally invasive percutaneous cystostomy with ureteroscopic pneumatic lithotripsy is a safe, efficient and easy treatment for calculi in bladder diverticula. This method provides a new clinical approach for lithotripsy and we suggest that it is worthy of wider use.
